# Integrative bioinformatics and validation studies reveal KDM6B and its associated molecules as crucial modulators in Idiopathic Pulmonary Fibrosis

**DOI:** 10.3389/fimmu.2023.1183871

**Published:** 2023-05-19

**Authors:** Anning Chen, Zhun Sun, Donglin Sun, Meiying Huang, Hongwei Fang, Jinyuan Zhang, Guojun Qian

**Affiliations:** ^1^ Affiliated Cancer Hospital and Institute of Guangzhou Medical University, Guangzhou, China; ^2^ Department of Anesthesiology, Zhongshan Hospital, Fudan University, Shanghai, China; ^3^ Department of Pain, Shanghai East Hospital, Tongji University School of Medicine, Shanghai, China

**Keywords:** Idiopathic Pulmonary Fibrosis, chromatin-modifying enzymes, disease biomarker, hub genes, gene ontology, drug molecule

## Abstract

**Background:**

Idiopathic Pulmonary Fibrosis (IPF) can be described as a debilitating lung disease that is characterized by the complex interactions between various immune cell types and signaling pathways. Chromatin-modifying enzymes are significantly involved in regulating gene expression during immune cell development, yet their role in IPF is not well understood.

**Methods:**

In this study, differential gene expression analysis and chromatin-modifying enzyme-related gene data were conducted to identify hub genes, common pathways, immune cell infiltration, and potential drug targets for IPF. Additionally, a murine model was employed for investigating the expression levels of candidate hub genes and determining the infiltration of different immune cells in IPF.

**Results:**

We identified 33 differentially expressed genes associated with chromatin-modifying enzymes. Enrichment analyses of these genes demonstrated a strong association with histone lysine demethylation, Sin3-type complexes, and protein demethylase activity. Protein-protein interaction network analysis further highlighted six hub genes, specifically KDM6B, KDM5A, SETD7, SUZ12, HDAC2, and CHD4. Notably, KDM6B expression was significantly increased in the lungs of bleomycin-induced pulmonary fibrosis mice, showing a positive correlation with fibronectin and α-SMA, two essential indicators of pulmonary fibrosis. Moreover, we established a diagnostic model for IPF focusing on KDM6B and we also identified 10 potential therapeutic drugs targeting KDM6B for IPF treatment.

**Conclusion:**

Our findings suggest that molecules related to chromatin-modifying enzymes, primarily KDM6B, play a critical role in the pathogenesis and progression of IPF.

## 1 Introduction

Idiopathic Pulmonary Fibrosis (IPF) is a chronic and progressive lung disorder that is characterized by injuries to the alveolar epithelial cells, leading to anomalous epithelial repair, fibroblast buildup, and excessive deposition of extracellular matrix (1, 2). The pathogenesis of IPF is complex and involves complicated interactions between various cell types and signaling pathways. Despite considerable advancements in our understanding of IPF and the availability of different treatment strategies, the morbidity and mortality linked to IPF remain severe, accounting for approximately 20% of all cases of interstitial lung disease and affecting approximately 3 million individuals across the globe (3).

Chromatin-modifying enzymes regulate the chromatin structure *via* post-translational modifications, communication, and interaction between the enzymes (4). The most common chromatin modifications can be divided into 4 categories, such as DNA methylation, histone methylation, histone acylation/acetylation, and histone ubiquitination (5, 6). Histone tails possess different modified residues, and the post-translational histone modifications help in changing the chromatin structure. The Jumonji structural domain-containing protein-3 (KDM6B) is a histone demethylase that regulates H3K27me3 trimethylation. Histone HDAC4 helps in the TGFβ1-induced differentiation of myofibroblasts, which is a vital step in IPF pathogenesis (7).

Recent advancements in microarray technology have facilitated biological research. The mRNA databases that are derived using the microarray technology offer valuable data to identify pathogenic variables and also inspire further research (8–10). Even though chromatin-modifying enzymes play a vital role in the onset and progression of IPF, very little information regarding their effect on IPF is available. Therefore, it is imperative to study the correlation between chromatin-modifying enzymes and IPF pathogenesis.

In this study, the differentially expressed genes linked to chromatin-modifying enzymes were used to identify several important cellular signaling pathways and the gene networks linked to IPF pathogenesis. The potential application of KDM6B related molecules as a novel biomarker that could be used for targeted therapy in IPF patients was further highlighted. This study helped in screening the probable candidate KDM6B-targeting drugs, which could be used as an effective treatment strategy for IPF. In summary, this study offers novel insights regarding the molecular mechanisms involved in IPF and presents strategies for developing biomarkers and therapies for treating this debilitating disease.

## 2 Materials and methods

### 2.1 Microarray data source

**Figure 1:**
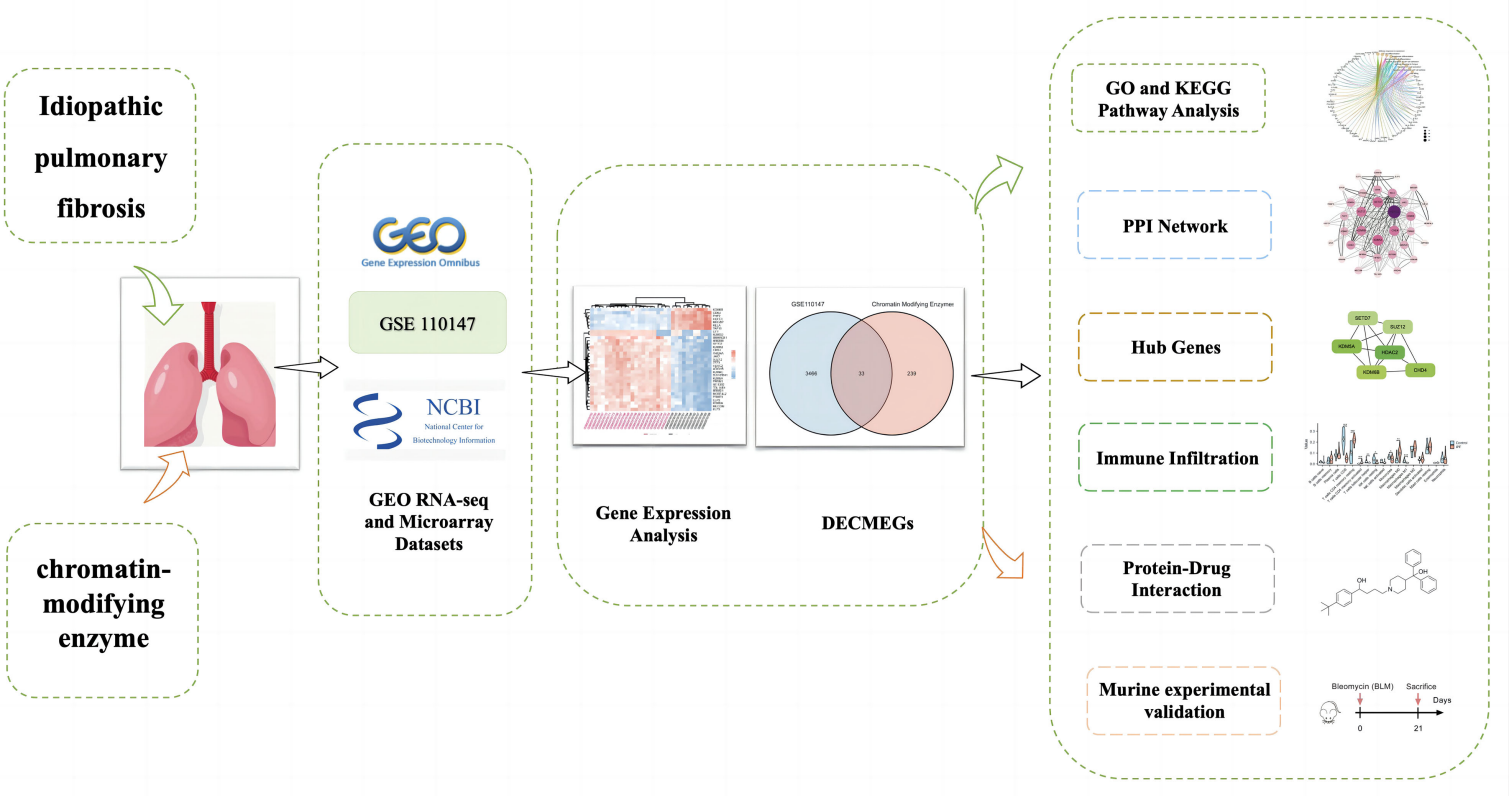
Schematic representation of the workflow used in this study.

**Figure 1** presents the analytical process used in this study. The GSE110147 dataset was retrieved from the GEO database, and it included 48 samples for RNA expression analysis (11). This dataset included 11 normal lung tissue samples, 10 patients with non-specific interstitial pneumonia (NSIP), 22 IPF patients, and 5 patients having mixed IPF-NSIP (**Table 1**). This study primarily focused on exploring the 22 IPF and 11 normal lung tissue samples that were retrieved from the GSE110147 dataset.

**Table 1:** Details of the GEO IPF data.

Dataset	Platform	Number of samples (IPF/control)
GSE110147	GPL6244	33 (22/11)
GSE33566 GSE156310	GPL6480 GPL18573	123(93/30) 21

GEO, Gene Expression Omnibus; IPF, Idiopathic pulmonary fibrosis.

### 2.2 Identifying differently expressed chromatin-modifying enzyme related genes

In the IPF samples, we identified differentially expressed genes (DEGs) using the GEO2R tool (12) with the Benjamini-Hochberg correction to control for false discovery rate, with a threshold of |log2 fold change (FC)| > 1 and adjusted *P*-values (*P* adj) < 0.05. The *P* adj is a modified *P*-value used in multiple hypothesis testing, which improves control of false positive rates (13). The Gene Set Enrichment Analysis (GSEA) (14) database was utilized to retrieve the 272 genes associated with chromatin-modifying enzymes (CMERGs) (listed in **Supplementary Table S1**). The intersection between DEGs and CMERGs, referred to as DECMEGs, represented the genes associated with chromatin-modifying enzymes and showed differential expression in the IPF samples.

### 2.3 GO, KEGG, and DO enrichment analyses of DECMEGs

In our study, we utilized the “clusterProfiler” package (15) in R to identify potential functions and pathways associated with the DECMEGs. We conducted Gene Ontology (GO) and Kyoto Encyclopedia of Genes and Genomes (KEGG) pathway analyses, employing an enrichment factor and a standardized metric (*P*-value < 0.05, *Q*-value < 0.25) to prioritize the most relevant functional items and pathways. The enrichment factor represents a statistical method employed to ascertain whether a set of genes (e.g., upregulated genes under specific conditions) exhibit overrepresentation or underrepresentation of particular GO/KEGG terms, based on their annotations (16, 17). The EnrichR online platform (https://maayanlab.cloud/Enrichr/) was utilized for DO enrichment analysis, and disease tool was used to enrich for diseases associated with IPF (18).

### 2.4 Protein-protein interaction network and module analyses

The STRING tool (19) was employed to investigate the protein-protein interaction (PPI) network using proteins encoded by DECMEGs (the identified differentially expressed genes associated with chromatin-modifying enzymes in IPF). The resulting PPI network was constructed, processed, and analyzed using Cytoscape (version 3.7.1) (20). The molecular complex detection (MCODE) plug-in facilitated module analysis within the PPI network. Genes exhibiting significant correlations in candidate modules are denoted as hub genes (21). To identify hub genes within the PPI network, the cyto-Hubba program was utilized, followed by Gene Ontology enrichment analysis using the ClueGO plug-in.

### 2.5 Immune infiltration analysis

Immune cell infiltration in control and IPF groups was evaluated using CIBERSORT, a deconvolution algorithm employing linear support vector regression for precise quantification of 22 immune cell types within gene expression profiles (22). This approach, also known as “digital cytometry,” has demonstrated a strong correlation with flow cytometric analysis results (23).

Associations among immune cells and between hub genes and immune cells were analyzed using GraphPad Prism 8.0.2 software (24). Furthermore, the proportions of each immune cell type in IPF tissue samples and healthy control samples were determined.

### 2.6 Exploring the ceRNA network of hub genes

The ceRNA network analysis was conducted for assessing the miRNA-mRNA interactions. The TargetScan (25), miRNet (26), and miRWalk (27) databases were used to identify probable miRNAs that target hub genes. If the same data was retrieved from every database simultaneously, accurate results were produced. LncRNAs that may target miRNA were predicted using the miRNet database and compared with the differentially expressed lncRNAs in the IPF. lncLocator was used to predict the subcellular localization of the lncRNAs (28). Xiantao Academy tool (https://www.xiantao.love/) is used for relevant statistical analysis and data visualization.

### 2.7 Gene set enrichment analysis

For GSEA analysis (14), the GSEA software (https://www.gseamsigdb.org/gsea/index.jsp) was downloaded and the samples were classified into the high (≥50%) and low (<50%) expression groups, based on the KDM6B expression levels. Furthermore, the c2.cp.v7.4.symbols.gmt subset was also downloaded from the Molecular Signatures Database to assess the important pathways and the molecular mechanisms, depending on the phenotype groups and gene expression profiles. A minimum of 5 gene sets and a maximum of 5, 000 gene sets were used, with 1, 000 resamplings. Furthermore, values with *P*-values < 0.05 and *FDR* < 0.25 were deemed statistically significant.

### 2.8 Analysis of protein subcellular localization and correlation with immune checkpoints

The Cell-PLoc 3.0 software (29), which includes a collection of web servers to predict the subcellular protein localization in various animals, was used to predict the subcellular localization of the KDM6B protein molecules. The relationship between KDM6B and key immune checkpoints (30) like CTLA4, PD-1, PDL2 was examined using the Pearson’s correlation coefficient from the GraphPad Prism 8.0.2 software.

### 2.9 Drug-gene interaction

The DSigDB database was used to assess the different drug-gene interactions and determine the important pharmacological compounds. Furthermore, the PubChem (31) and PDB (32) databases were searched to identify the molecular structures of different ligands and target proteins.

### 2.10 Mouse model

Male mice (C57BL/6, 8-week-old, 22–25 g) were randomly classified into two different groups (i.e., bleomycin and control groups). The murine pulmonary fibrosis model was constructed by intratracheal injection of bleomycin (5 mg/kg). Mice were euthanized 21 days after injection. The Animal Experimentation Committee at Guangzhou Medical University approved all the animal experiments (project number: Casgene-2022120100559). The experiments were conducted following the guidelines for handling and using laboratory animals.

Lung RNA was extracted using an RNA isolation kit (Tiangen) and subsequently reverse transcribed (Tiangen). Actin functioned as the internal control, and the relative gene expression was calculated using the ΔΔCt quantification method.

### 2.11 Western blot analysis

The lung tissues were lysed with a strong RIPA lysis solution containing protease inhibitors, phosphatase inhibitors, and EDTA (pH 8.0). The cell lysate was incubated on ice for 30 mins, centrifuged, and clarified, and the cell-free supernatant was collected. The BCA protein assay kit was used to determine the protein concentrations. Use a 12.5% concentration of protein gel with a protein loading volume of 15μg. The different proteins in the samples were electrophoresed using the SDS/PAGE tand transferred to PVDF membranes. The PVDF membranes were blocked using a non-fat milk solution (5%) in TBST and incubated in the presence of a primary antibody (Mouse monoclonal/Rabbit monoclonal). Thereafter, the membranes were incubated with the secondary antibodies (Goat Anti-Rabbit/Rabbit Anti-Mouse) and rinsed with TBST, and the protein bands were observed using an ECL reagent and a Tanon Imager.

### 2.12 Construction and validation of the diagnostic model

A diagnostic model was constructed using six hub genes (KDM6B, KDM5A, SETD7, SUZ12, HDAC2, and CHD4) and the IPF characteristic gene TGF-β1 in the GSE110147 dataset (33). The model was presented as a nomogram, and its clinical diagnostic value was assessed using calibration curve (CC) analysis, decision curve analysis (DCA), and clinical impact curve (CIC) analysis.

To ensure the reliability of this diagnostic model, the GSE33566 (34) dataset was employed as a validation set. Subsequently, receiver operating characteristic (ROC) curve analysis (35) was performed to evaluate the diagnostic value of the nomogram.

### 2.13 Single-cell data mining and analysis

The single-cell RNA sequencing (scRNA-seq) data from human IPF samples were obtained from the GSE156310 dataset (36) in the GEO. The GSE156310 dataset comprises scRNA-seq data derived from 21 explant lung tissue specimens, which were obtained from patients with advanced IPF, systemic sclerosis-associated interstitial lung disease, and organ donor controls. Computational analysis of the GSE156310 dataset was conducted using the R package “Seurat” (version 4.0.3) (37). Principal component analysis (PCA) was executed with the Seurat RunPCA() function, while scRNA-seq data normalization was performed using the Seurat NormalizeData() function. The Seurat FindIntegrationAnchors() and IntegrateData() functions, based on robust principal component analysis (RPCA), were employed to integrate multiple samples. Dimension reduction was carried out using t-distributed stochastic neighbor embedding (tSNE), and Louvain clusters were calculated using the first 30 principal components with the RunUMAP function. Cell annotations were made using a combination of the BP and HPCA databases. Gene expression and distribution were visualized using the Seurat DotPlot(), VlnPlot(), and FeaturePlot() functions.

### 2.14 Statistical analysis

An unpaired Student’s *t*-test was conducted to analyze the data derived from both groups. Additionally, Pearson’s correlation coefficient was conducted to assess any possible association between the variables. All statistical analysis was carried out using the GraphPad Prism and R software (ver. 4.2.0), where values with *P <0.05* were deemed significant.

## 3 Results

### 3.1 Identifying the DECMEGs in IPF

**Figure 2:**
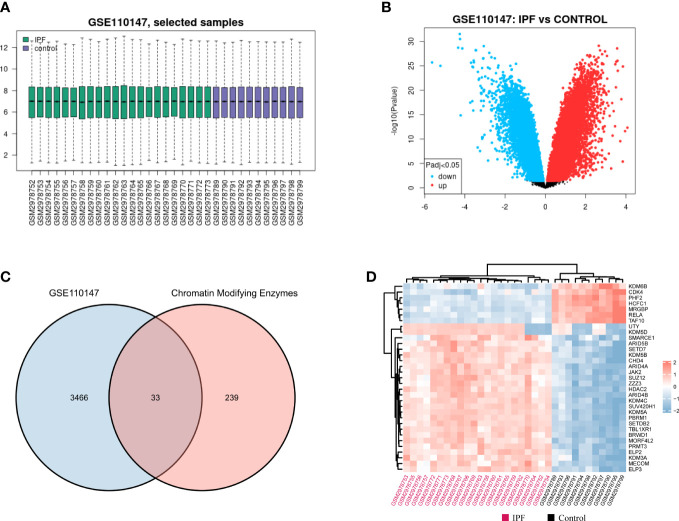
Identifying the DECMEGs in IPF. **(A)** Normalization of the samples selected from the GSE110147 dataset. **(B)** The DEGs identified from the GSE110147 dataset. **(C)** The DECMEGs of IPF. **(D)** The heatmap of the DECMEGs.

The normalized gene expression profile dataset for IPF (GSE110147) has been depicted in **Figure 2A**. The 3, 499 DEGs that were detected in the GSE110147 dataset were further presented using a volcano plot (**Figure 2B**). **Figure 2C** displays the 33 consistent DECMEGs that were identified by the integrated bioinformatics analysis. **Figure 2D** displays the heatmap of the DECMEGs.

### 3.2 Functional enrichment analysis of DECMEGs

**Figure 3:**
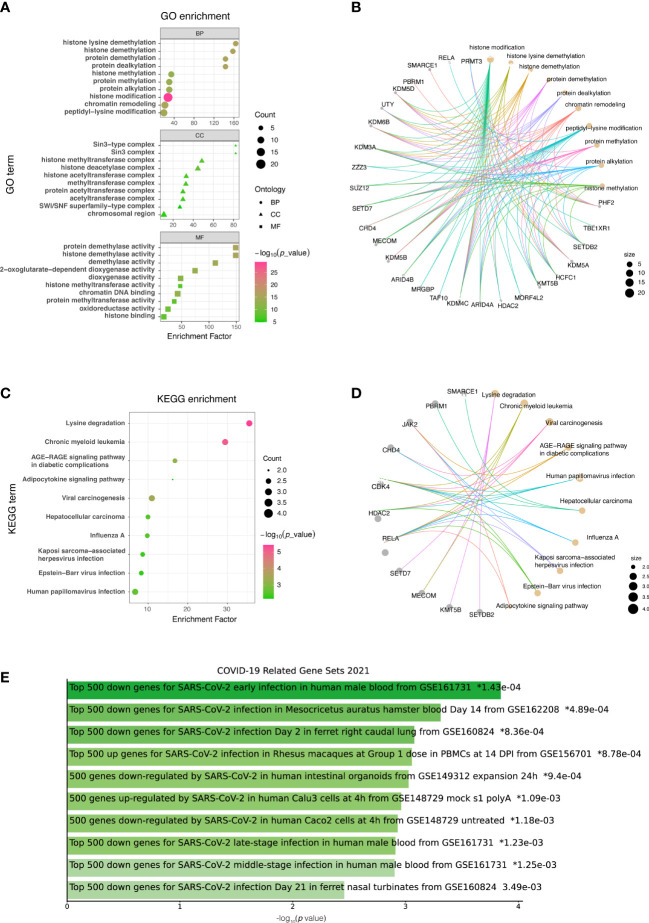
List of DECMEGs for functional enrichment analysis. **(A)** GO. **(B)** GO analysis network diagram. **(C)** KEGG. **(D)** KEGG analysis network diagram. **(E)** DO enrichment, green horizontal bar represents the items with valid P-values (<0.05).

GO analysis was conducted to determine the biological functions of DECMEGs. The findings revealed that these genes were primarily involved in histon lysine demethylation (BP), Sin3-type comlex (CC), and protein demethylase activity (MF) (**Figures 3A, B**). KEGG pathway analysis revealed that the lysine degradation was primary involved (**Figures 3C, D**), and it was also enriched and significantly linked to COVID-19 (**Figure 3E**).

### 3.3 PPI network and hub gene analysis

**Figure 4:**
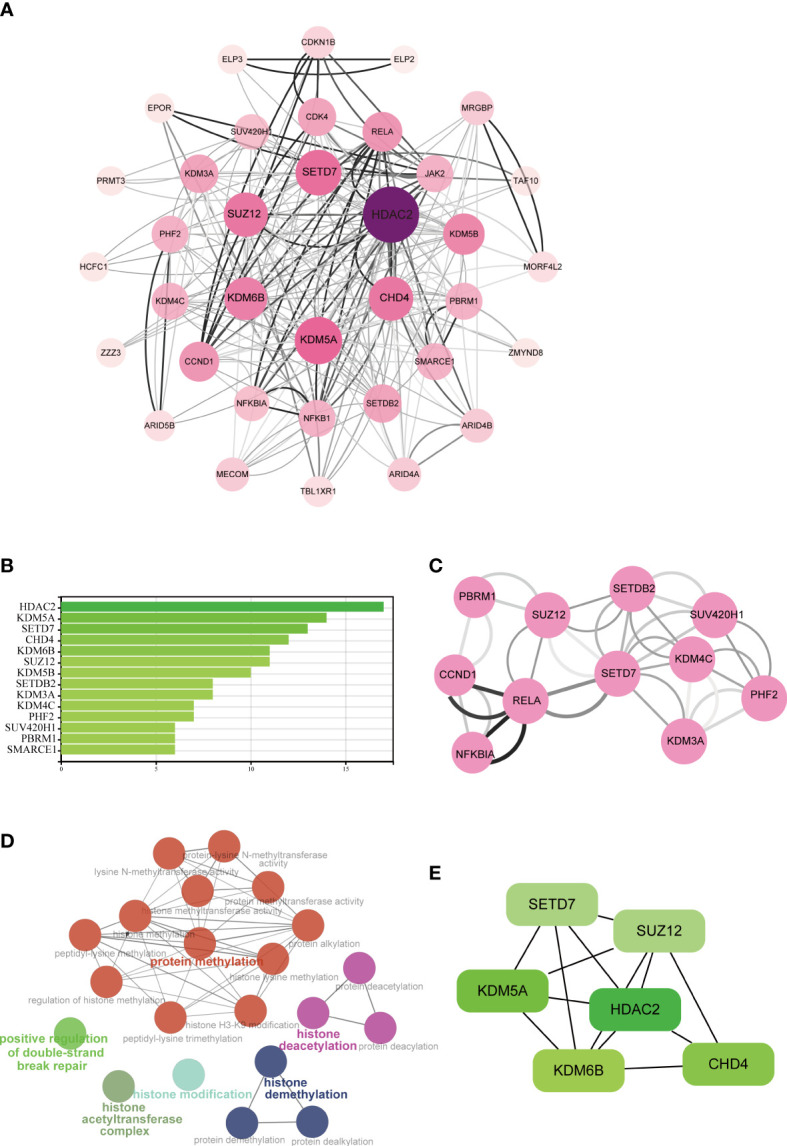
Analysis of the PPI networks and hub genes. **(A)** PPI networks of DECMEGs, where bigger edge and node sizes imply higher degrees. **(B)** Connectivity ranks of the genes. **(C)** The primary module in the PPI network. **(D)** Biological processes of hub genes analyzed by ClueGO tool. **(E)** The six hub genes of IPF.

The STRING database was used to construct a PPI network for identifying the links between the DECMEGs (**Figure 4A**). A majority of the connected nodes were identified as hub genes. According to **Figure 4B**, the target links in the PPI network were ranked in ascending order, from the smallest to the largest. The most significant module with 14 edges and 8 nodes was chosen in this study (**Figure 4C**). The role of hub genes was further examined using the ClueGO plugin, as the genes closer in the network were seen to be more fundamentally regulated. **Figure 4D** shows that they are predominantly enriched in processes such as protein methylation, histone deacetylation, histone modification, and histone demethylation. The six genes with the highest Maximum Clique Centrality (MCC) score, such as KDM6B, KDM5A, SETD7, SUZ12, HDAC2, and CHD4, were regarded as the IPF hub genes (**Figure 4E**, **Table 2**).

**Table 2:** The top 6 hub genes.

Genes	Description	Degree	MCC	MNC	Stress	Log2FC	Expression change
**KDM6B**	lysine demethylase 6B	11	636	11	208	1.0378764	Upregulated
**KDM5A**	lysine demethylase 5A	14	615	13	354	-1.9576636	Downregulated
**SETD7**	SET domain containing lysine methyltransferase 7	13	515	12	428	-1.1817614	Downregulated
**SUZ12**	SUZ12 polycomb repressive complex 2 subunit	11	278	11	198	-1.01827	Downregulated
**HDAC2**	histone deacetylase 2	17	191	16	710	-1.3501845	Downregulated
**CHD4**	chromodomain helicase DNA binding protein 4	12	44	10	312	-1.1376968	Downregulated

MCC, maximal clique centrality; MNC, maximum neighborhood component.

### 3.4 Immune infiltration analysis

**Figure 5:**
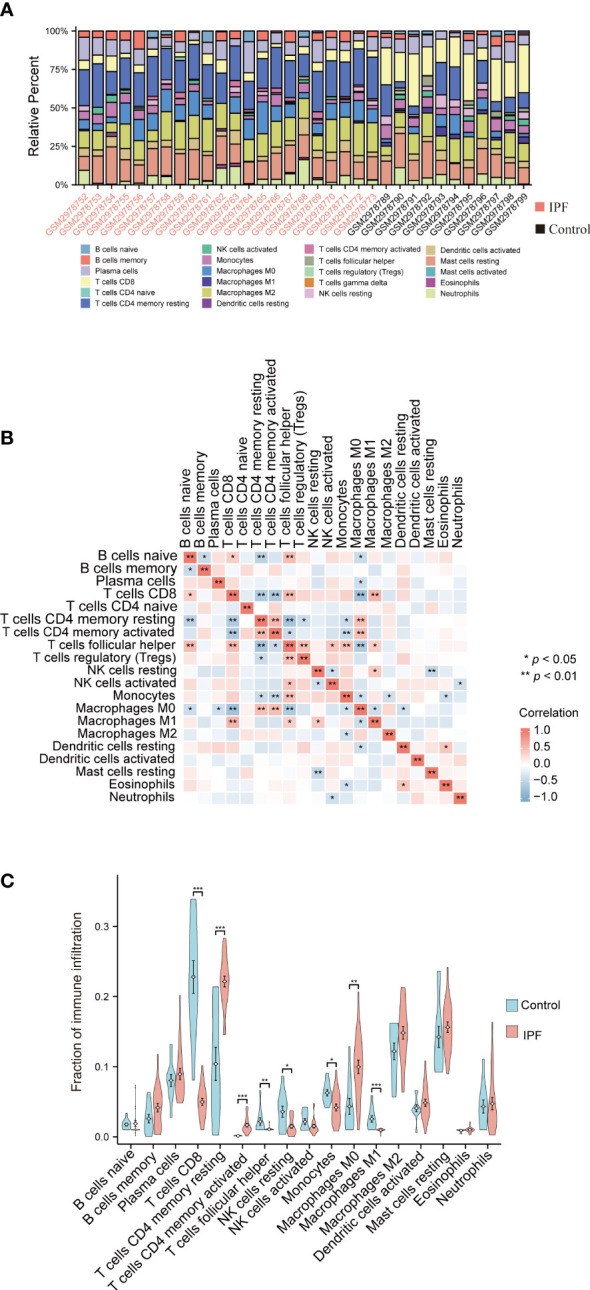
Analysis of the immune infiltration levels in the IPF. **(A)** The ratio of 22 immune cells in each IPF sample. **(B)** The relationship between each immune cell. **(C)** The proportion of immune cells in the control and IPF samples. *P < 0.05, **P < 0.01, ***P < 0.001.

The proportions of the immune cells in the IPF tissue and control samples were analyzed using the CIBERSORT method to evaluate the IPF immune cell composition (**Figure 5A**). Multiple types of immune cells, including naive and memory B cells, resting memory CD4^+^ T cells, CD8^+^ T cells, follicular helper T cells, and M0 macrophages showed a strong association (**Figure 5B**). The results showed that IPF tissue had a lower proportion of CD8^+^ T cells, follicular helper T cells, resting NK cells, and M1 macrophages compared to the control group and a higher proportion of activated memory CD4^+^ T cells, resting memory CD4^+^ T cells, and M0 macrophages (**Figure 5C**).

**Figure 6:**
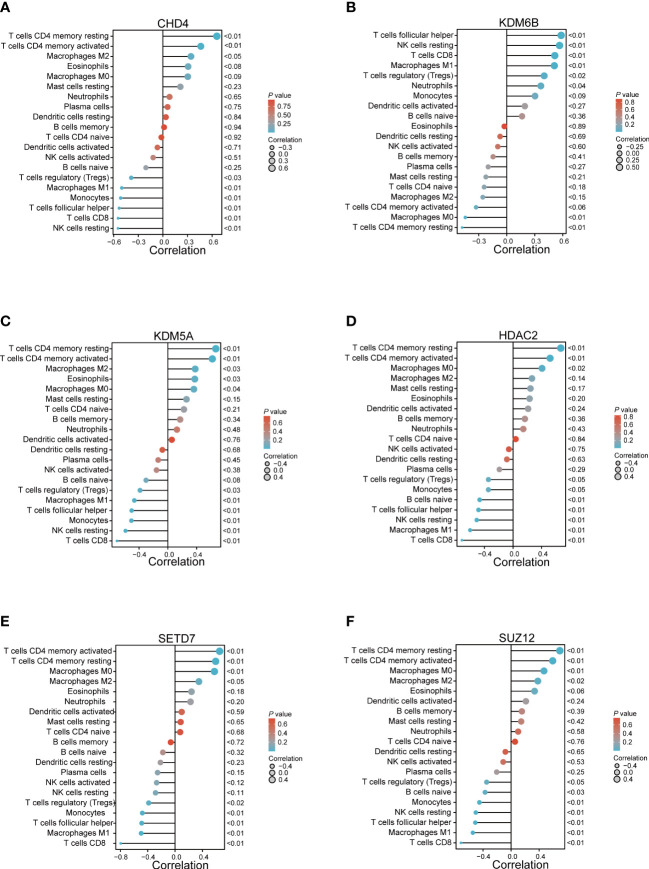
The relationship between the immune cells and hub genes. **(A)** CHD4; **(B)** KDM6B; **(C)** KDM5A; **(D)** HDAC2; **(E)** SETD7; and **(F)** SUZ12.

The Pearson correlation coefficient was then used to assess the relationship between the abundant immune cells and the expression of hub genes. The findings demonstrated a negative relationship between CD8^+^ T cells and CHD4, KDM5A, HDAC2, SETD7, and SUZ12, but a positive relationship was observed between CD8^+^ T cells and KDM6B. Similarly, a positive relationship was observed between the resting and activated memory CD4^+^ T cells and CHD4, HDAC2, KDM5A, SETD7, and SUZ12, but it was negatively correlated with KDM6B (**Figure 6**).

### 3.5 The mRNA–miRNA–lncRNA ceRNA network of IPF

**Figure 7:**
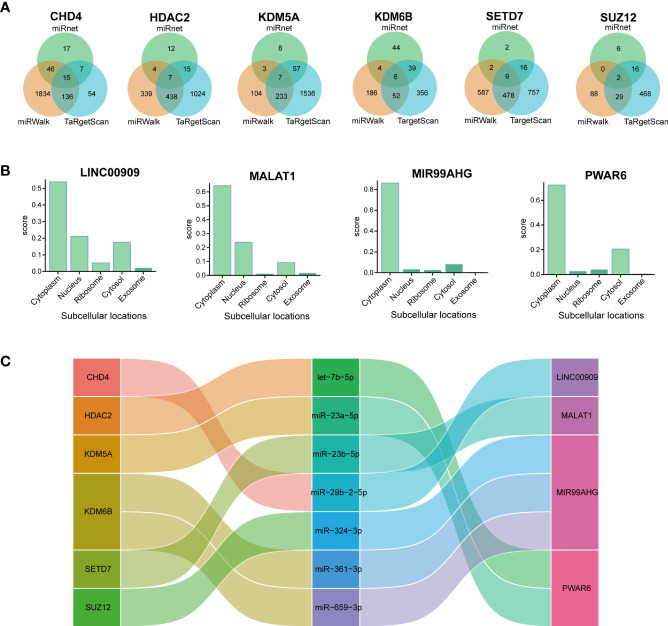
Constructing the lncRNA–miRNA–mRNA ceRNA IPF network. **(A)** Venn diagram presents the miRNAs that target each hub gene. **(B)** The subcellular localization of lncRNAs of ceRNA. **(C)** The alluvial diagram presents the ceRNA network.

Although non-coding RNAs (ncRNA) do not encode proteins, they participate in numerous biological processes. The interplay within the mRNA-miRNA-lncRNA ceRNA network offers valuable insights into the regulatory mechanisms underlying pulmonary fibrosis (38). All object databases simultaneously acknowledged the miRNA-targeting hub gene as a valid gene, and **Figure 7A** presents their Venn diagrams. Additionally, the intersections between this miRNA and 359 DElncRNAs were predicted using the miRNet online database. The ceRNA mechanism suggested that the expression patterns of mRNAs and lncRNAs in the ceRNA network should be consistent. The analysis retrieved seven distinct DElncRNAs, which act as ceRNAs, regulating the expression of their target mRNAs in the cytoplasm and competing with miRNAs. The lncLocator predicted that only four DElncRNAs (MIR99AHG, MALAT1, PWAR6, and LINC00909) were located in the cytoplasm (**Figure 7B**). A network of ceRNAs was identified, including CHD4/miR-29b-2-5p/LINC00909, HDAC2/let-7b-5p/PWAR6, and KDM5A/miR-23a-5p/PWAR6 (**Figure 7C**).

### 3.6 GSEA of KDM6B

**Figure 8:**
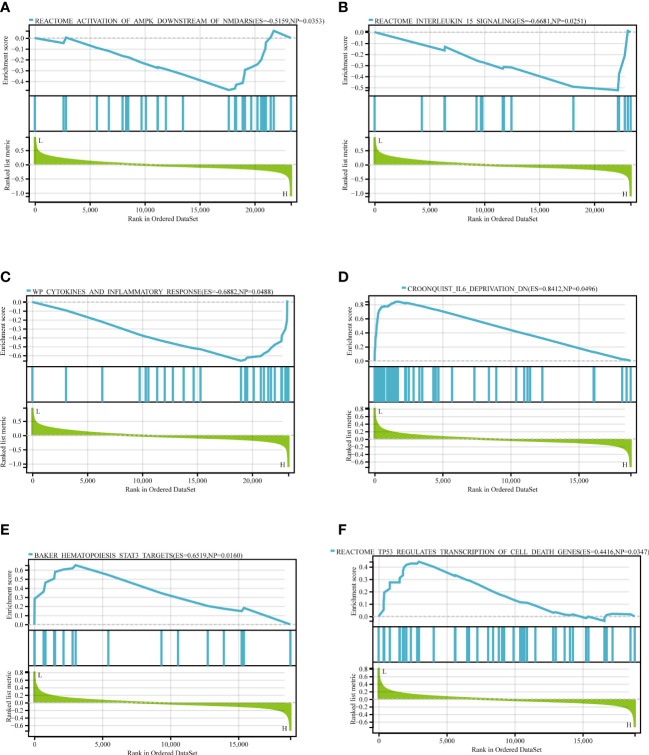
The GSEA of KDM6B. **(A)** Reactome activation of AMPK downstream of NMDARs. **(B)** Reactome IL-15 signaling pathway **(C)** WP cytokines and inflammatory response. **(D)** Croonquist IL-6 deprivation DN. **(E)** Baker hematopoiesis of STAT3 targets. **(F)** TP53 regulates the transcription of cell death genes.

The KDM6B gene exhibited the largest MCC among the hub genes as demonstrated in **Table 2**. In addition, **Figures 6** and **7** demonstrated the significance of KDM6B in defining the immune infiltration and ceRNA networks in IPF, making it an ideal candidate for additional research. GSEA results further validated the crucial role of KDM6B in the immunological infiltration and ceRNA networks of IPF. The analysis revealed that KDM6B was significantly enriched in several signaling pathways, including activation of AMPK downstream of NMDARs, IL-15 signaling, cytokines and inflammatory response, IL-6 deprivation DN, STAT3 targets in hematopoiesis, and regulation of cell death gene transcription by TP53 (**Figure 8**).

### 3.7 Subcellular localization of the protein and its relationship with the immune checkpoint analyses of KDM6B

**Figure 9:**
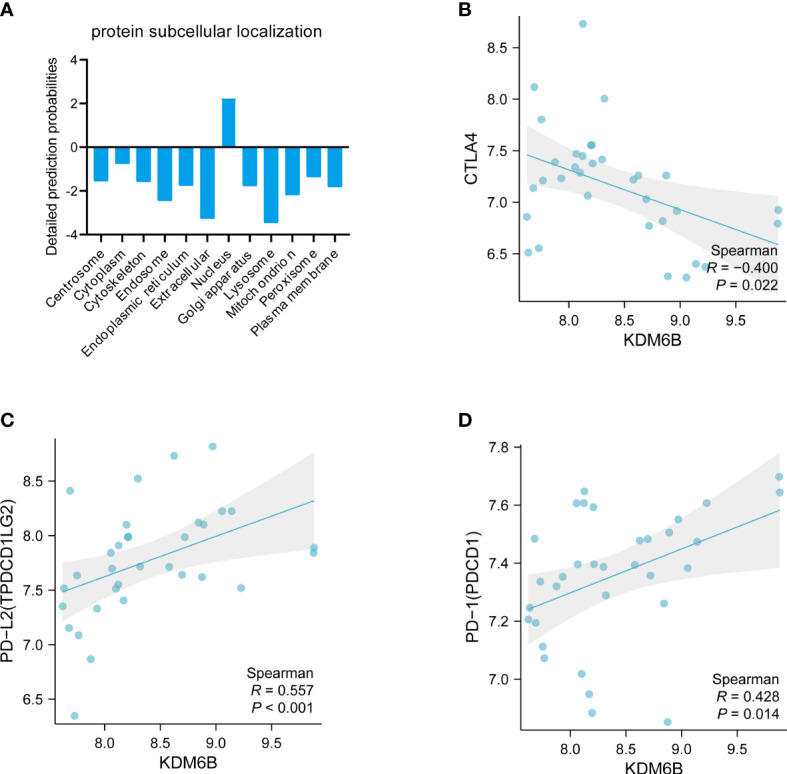
Comprehensive analysis of KDM6B. **(A)** Subcellular localization of the KDM6B protein. Relationship between KDM6B and immune checkpoints such as **(B)** CTLA4; **(C)** PD-L2; **(D)** PD-L1.

The subcellular localization of proteins plays a significant role in determining their biological functions (39). According to Cell-PLoc 3.0 analysis, KDM6B protein is predicted to be localized in the nucleus (**Figure 9A**), which is consistent with a previous study (40). **Figures 9B–D** showed that KDM6B was strongly correlated with key immune checkpoints, such as CTLA4, PDL2, and PD-1, further emphasizing the vital role played by KDM6B in regulating the immune response in IPF.

### 3.8 Drug-gene interactions analysis of KDM6B

An innovative therapy strategy involves the utilization of potential therapeutic drugs that target KDM6B. **Table 3** displays the drug-gene interaction network for KDM6B. The Enrichr tool revealed 39 promising therapeutic drug candidates using the transcriptional signature from the DSigDB database, and the 32 leading candidates were chosen according to their *P-values*. The top 10 enriched pharmaceuticals in the DSigDB database have been presented in **Table 3**.

**Table 3:** Drug-gene interaction network of KDM6B.

Name	*P-value*	Chemical formula	Structure
primaquine HL60 UP	0.00149996975933348	C_15_H_21_N_3_O	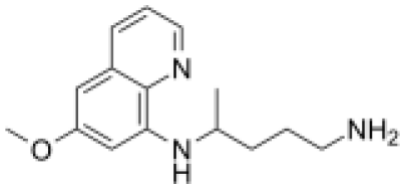
gossypol HL60 UP	0.00359994565021312	C_30_H_30_O_8_	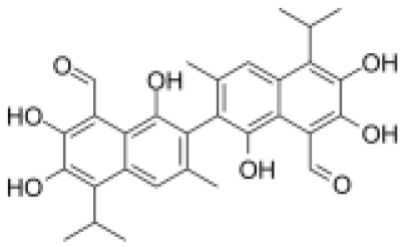
azacitidine MCF7 UP	0.0051499318302617	C_8_H_12_N_4_O_5_	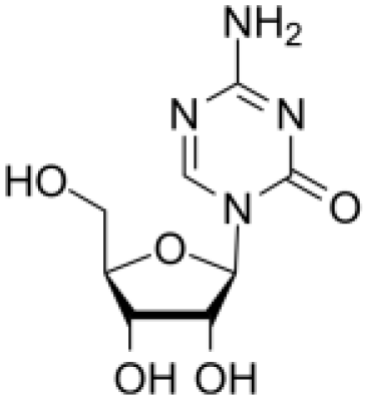
niclosamide MCF7 UP	0.00614992398429618	C_13_H_8_C_l2_N_2_O_4_	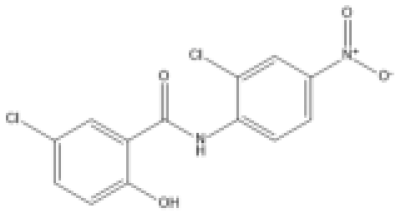
pyrvinium HL60 UP	0.00684991887976516	C_26_H_28_N_3_^+^	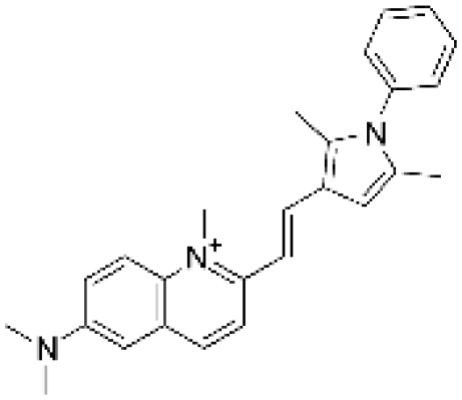
terfenadine MCF7 UP	0.00919990356484476	C_32_H_41_NO_2_	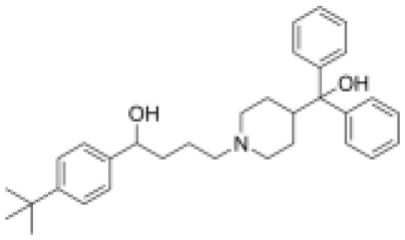
helveticoside HL60 UP	0.015049873627526	C_29_H_42_O_9_	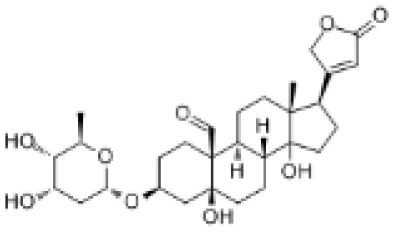
8-azaguanine PC3 UP	0.0161498689111765	C_4_H_4_N_6_O	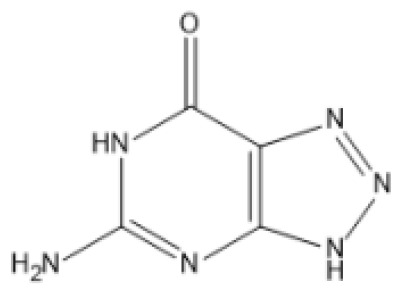
proscillaridin HL60 UP	0.0161498689111765	C_30_H_42_O_8_	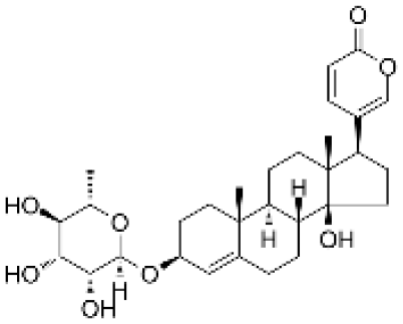
strophanthidin HL60 UP	0.0171998646182966	C_23_H_32_O_6_	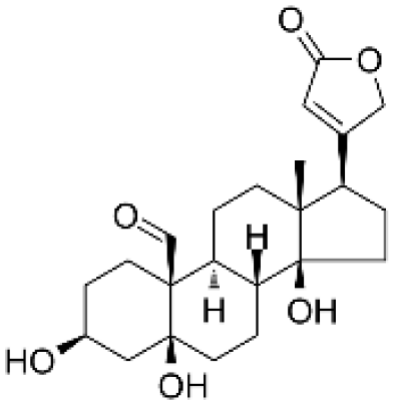

### 3.9 KDM6B may be involved in the development of IPF

**Figure 10:**
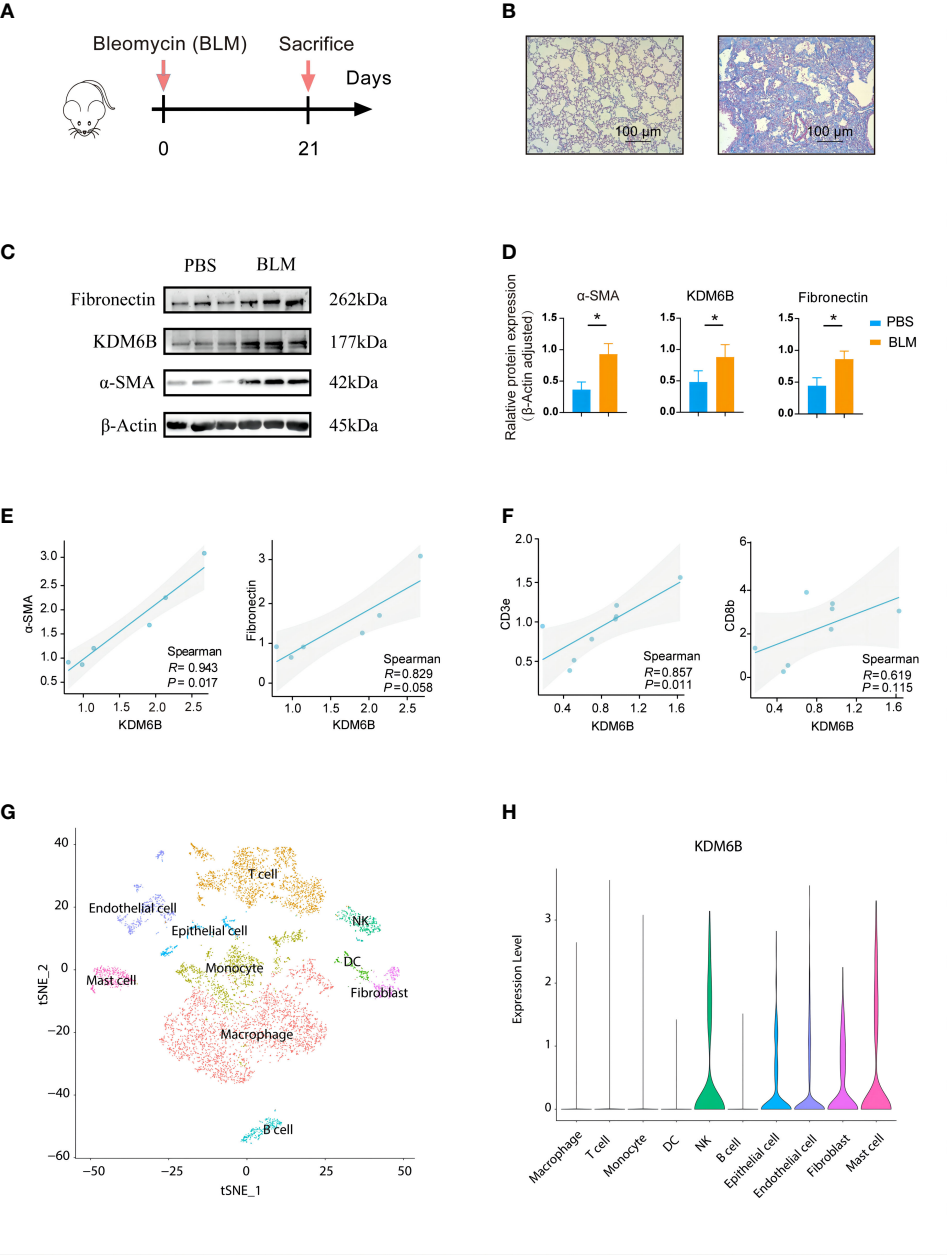
Validation of identified hub genes using a bleomycin-induced mouse model of pulmonary fibrosis. **(A)** Schematic diagram of the experimental protocol used in the bleomycin (BLM)-induced lung fibrosis model in mice. **(B)** Representative MASSON-stained lung sections. **(C)** Expression of fibrosis-associated proteins and KDM6B was determined by Western Blot. **(D)** Semi-quantitative b-Actin of the Western Blot method. **(E)** Correlation between KDM6B and a-SMA and fibronectin. **(F)** Correlation between KDM6B and CD3e and CD8b. **(G)** Visualizing single-cell clustering results using t-SNE plots. **(H)** Analyzing KDM6B gene expression across diverse lung cell subsets. *P < 0.05 Results are expressed as mean ± SEM.

Subsequently, experiments were conducted to explore the potential of KDM6B as a biomarker or therapeutic target for predicting IPF. Murine models of bleomycin-induced pulmonary fibrosis mouse model were employed (**Figures 10A, B**). KDM6B expression, along with macrophages, CD3^+^ and CD4^+^ T cells, was significantly elevated in the lungs of bleomycin-induced pulmonary fibrosis mice compared to healthy controls (**Figures 10C, D**, **S1A, B**). The results demonstrated a significant positive correlation between KDM6B and key pulmonary fibrosis proteins, such as fibronectin and α-SMA (**Figures 10C–E**). Additionally, our RT-PCR results revealed a statistically significant positive correlation between KDM6B and CD3 and CD8 (**Figure 10F**), while KDM6B expression exhibited a negative correlation with CD4, albeit not statistically significant (**Figure S1C**). These findings support our bioinformatics results. Furthermore, by analyzing publicly accessible single-cell RNA sequencing data of human IPF (GSE156310), we determined that KDM6B was predominantly expressed in natural killer (NK) cells, epithelial cells, endothelial cells, fibroblasts, and mast cells within the lung (**Figures 10G, H**). Collectively, these results suggest that KDM6B may play a critical role in IPF pathogenesis.

### 3.10 Construction of a diagnostic model using KDM6B

**Figure 11:**
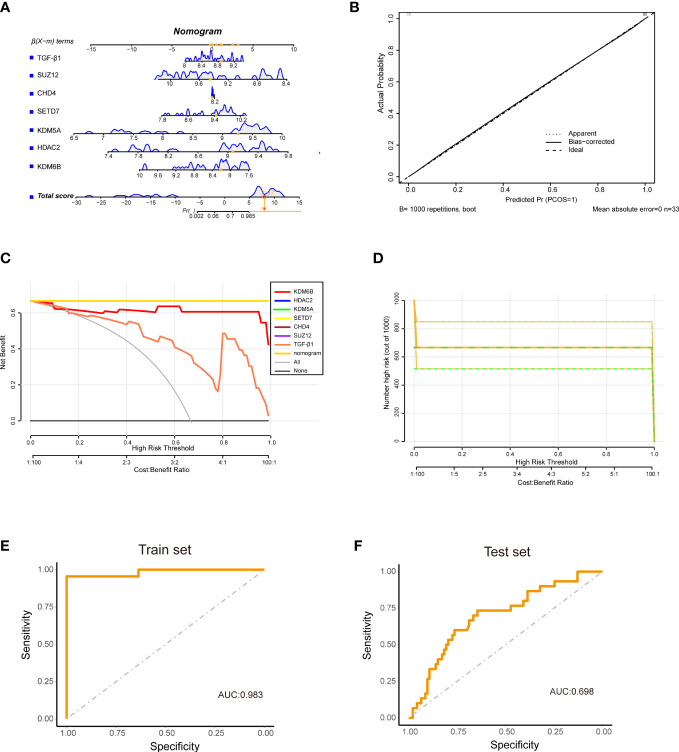
Construction of a diagnostic model using hub Genes and TGF-β1. **(A)** The diagram illustrates a diagnostic model built upon six hub genes in conjunction with TGF-β1. **(B)** Calibration curve **(CC)** representing the accuracy of the diagnostic model. **(C)** Decision curve analysis **(DCA)** illustrating the net benefit of the diagnostic model. **(D)** Clinical impact curve derived from the DCA, assessing the nomogram’s performance. **(E)** ROC curve for the training cohort (GSE110147). **(F)** ROC curve for the validation cohort (GSE33566).

Finally, the diagnostic model of IPF was constructed centered on the KDM6B and other hub genes we identified. The model was developed using multivariate logistic regression analysis and presented as a nomogram (**Figure 11A**). The nomogram showed a strong concordance between predicted and actual IPF and healthy control samples (**Figure 11B**). Furthermore, decision curve analysis demonstrated that patients could benefit from diagnostic models with a central gene threshold probability ranging from 0 to 1 (**Figures 11C, D**). The ROC curve analysis indicated that the diagnostic model had an area under the curve (AUC) of 0.983 (**Figure 11E**). To further validate the diagnostic model’s reliability, we utilized an external dataset (GSE33566) as a validation cohort, consisting of 93 IPF lung samples and 30 healthy control lung samples. The AUC was 0.698 (**Figure 11F**). These results suggest that our diagnostic model, which utilizes KDM6B, possesses a robust ability to differentiate IPF patients from healthy individuals.

## 4 Discussion

IPF is a lung-specific, chronic, fibrosing, and progressive disease with an unfavorable prognosis and an unidentified etiology (41). The exact pathogenesis of IPF is yet to be fully understood, but it is believed to result from a complex interplay of multiple cell types and signaling pathways (42). An important aspect of the pathogenesis of lung diseases includes the regulation of gene expression by chromatin-modifying enzymes (43). These enzymes play a crucial role in determining the appropriate stage-specific gene expression during immune cell development. However, the precise molecular mechanisms underlying the involvement of chromatin-modifying enzymes in IPF remain unclear and require further investigation. This study aims to identify and validate chromatin-modifying enzyme-related genes as potential key biomarkers for IPF, utilizing a bioinformatics and systems biology approach, with a focus on histone modifications.

In this study, transcriptome analysis was carried out on IPF patients to identify the DEGs. The findings of the transcriptome analysis revealed 3, 499 DEGs between the control and IPF groups, out of which 33 DECMEGs were identified. Further analysis of the biological significance of DECMEGs showed that they are mainly involved in histone-lysine demethylation, Sin3-type assemblies and protein demethylation enzyme activity, and that protein demethylation enzyme activity is critical for IPF pathogenesis (44). Our DO analysis reveals a strong link between IPF and COVID-19, supporting prior findings (45, 46). The incidence of fibrotic lung disease post-SARS-CoV-2 infection is expected to be substantial, leading to a considerable global increase (47). Patients with IPF, who exhibit prevalent risk factors and reduced pulmonary reserve, may face a more unfavorable prognosis compared to the general population.

PPI networks and modular analysis were used to identify the six hub genes, including KDM6B, KDM5A, SETD7, SUZ12, HDAC2, and CHD4, which play a role in histone modification. Analyzing the immune infiltration in IPF tissue revealed a correlation between memory-activated CD4^+^ T cells, CD8^+^ T cells, and macrophages. Furthermore, the hub genes and the major invading cells in IPF were significantly correlated, particularly KDM6B is positively correlate with CD8^+^ T cells but negatively correlated with CD4^+^ T cell.

Although the pathogenesis of IPF is still not fully understood, T cells have been identified as contributors to fibrosis progression, and the underlying mechanisms are complex. Papiris et al. demonstrated a significant increase in CD8^+^ T cells in the lung tissues and bronchial lavage fluid of IPF patients. Furthermore, CD8^+^ T cells are associated with dyspnea grade and functional disease severity parameters. Regarding CD4^+^ T cells, the expression of chemokine receptors CXCR1 and CCR2 suggests that Th2 cells may predominate in IPF (48). Previous studies have shown that CD4^+^ cells in IPF are highly activated and exhibit exuberant responses when stimulated with autologous IPF lung extracts, suggesting a process of autoimmunity in IPF through recognition of self-antigens (49). These findings highlight the complex pathways by which T cells may regulate fibrosis. Our results suggest that KDM6B may play a critical role in regulating T cell response during IPF progression. Furthermore, given the low expression of KDM6B in T cells, as unveiled by our single-cell RNA analysis, it can be postulated that KDM6B potentially regulates T cell responses during IPF progression via an indirect mechanism.

In this study, a novel ceRNA network was constructed based on seven interactions, including the axis of KDM6B/miR-361-3p/MIR99AHG and SETD7/miR-23b-5p/MALAT1, to explore the regulatory mechanisms in IPF. The MIR99AHG/miR-136-5p/USP4/ACE2 signaling axis controls lung fibrosis and epithelial-to-mesenchymal transition, which prevents the progression of lung cancer to lung adenocarcinoma (50). MALAT1 is a key long-stranded non-coding RNA that plays a vital role in lung diseases (51). In conclusion, the DEmRNA–miRNA–DeIncRNA–ceRNA network is involved in modifying the IPF related chromatin-modifying enzymes.

The GSEA analysis found that KDM6B primarily participates in the activation of AMPK downstream of NMDARs, IL-15 signaling, WP cytokines, and inflammatory response, whereas TP53 regulates the transcription of cell death genes. Rangarajan et al. observed that both IPF patients and experimental murine models of pulmonary fibrosis exhibit the presence of metabolically-active and apoptosis-resistant myofibroblasts in the fibrotic regions, which is associated with a lower AMPK activation (52). Additionally, with IPF progression, patients tend to exhibit an accumulation of M2 macrophages, which secrete a variety of cytokines that promote the conversion of fibroblasts into myofibroblasts. The activation of the JAK2/STAT3 signaling pathway is shown to be important for the polarization of M2 macrophages (53). Research has identified TP53 target 1 (TP53TG1), a p53-inducible long non-coding RNA, as a dysregulated critical gene in the IPF regulatory network and a major downregulated gene in IPF-driven fibroblasts (54). In summary, several pathways enriched to KDM6B by GSEA analysis are closely associated with IPF.

Histone modifications play a significant role in regulating cell fate determination, terminal differentiation, and cellular inactivation (55, 56). KDM6B, a histone demethylase, is identified as the major regulator of different physiological processes, such as cell growth, differentiation, senescence, and inflammation (57–61). The human KDM6B (lysine-specific demethylase 6B) gene, which codes for a polypeptide with 1682 amino acids and an average molecular weight of 176 KDa, was localized on chromosome 17p13.1 (62, 63). Our analysis using the Cell-PLoc 3.0 tool predicted that KDM6B is located in the nucleus. Previous research has demonstrated that the subcellular distribution of KDM6B is stringently controlled by a dynamic equilibrium between nuclear import and export processes. Notably, the nuclear accumulation of KDM6B is a critical factor for the effective demethylation of H3K27me3 (64).

Our bioinformatics analysis further uncovered a strong correlation between KDM6B and well-established immune checkpoints, including CTLA4, PD-L2, and PD-L1, in IPF. This suggests that KDM6B may serve an immunomodulatory function in the treatment of IPF. While the relationship between immune checkpoints and IPF has not been explicitly reported in these studies, prior research has demonstrated that combining PD-1/PD-L1 and anti-CTLA4 inhibitors may enhance treatment efficacy, not only in non-small cell lung cancer but also in small cell lung cancer, thereby presenting a promising first- or second-line treatment option (65, 66). However, therapeutic choices for pulmonary fibrosis remain limited, with options such as immunosuppressive agents posing significant risks for elderly patients (67). Consequently, it is crucial to elucidate the relationship between immune checkpoints and IPF to better inform treatment strategies.

This study demonstrated increased KDM6B expression in the lungs of patients with IPF and in the bleomycin-induced pulmonary fibrosis mouse model, compared to healthy human lung tissue and control mice, respectively. Additionally, the pulmonary fibrosis-related proteins fibronectin and α-SMA exhibited strong correlations with KDM6B expression. We also developed a diagnostic model for IPF using KDM6B and other identified key genes. ROC curve analysis and statistical methods substantiated the model’s reliability, demonstrating its robust diagnostic value. Moreover, we discovered several novel therapeutic compounds targeting KDM6B, which may provide a promising new direction for IPF treatment.

This study presents persuasive findings while acknowledging certain limitations. We have partially validated KDM6B’s potential role in IPF through animal experiments and by constructing diagnostic and prognostic models using publicly available databases, highlighting KDM6B’s significance in IPF. Additionally, we identified KDM6B’s primary presence in NKs, epithelial cells, endothelial cells, fibroblasts, and mast cells, as supported by public scRNA-seq datasets. Nonetheless, to better understand KDM6B’s function and its associated molecules in IPF pathophysiology, generating a KDM6B knockout mouse model, part of our future research plans, would be beneficial. While CIBERSORT is a widely used and highly regarded “digital cytometry” method for accurately estimating immune cell infiltration, with strong correlations to flow cytometric analysis results, validating the prediction using flow cytometry is recommended. Furthermore, although we predict that MIR99AHG, MALAT1, PWAR6, and LINC00909 are located in the cytoplasm based on computational modeling, only MIR99AHG’s cytoplasmic localization was validated in a previous study (68). Therefore, conducting *in vitro* and *in vivo* experiments to further confirm our predictive outcomes is warranted. Moreover, the decision curve analysis indicates that the diagnostic weight of KDM6B is better than that of TGF-β1, but the curve of TGF-β1 is slightly steep, which may be caused by a small sample size. In future work, we will increase the sample size to further improve this study.

## 5 Conclusions

In summary, our study identified six hub genes, specifically KDM6B, KDM5A, SETD7, SUZ12, HDAC2, and CHD4, as critical regulators in IPF. Our animal experiments revealed that KDM6B expression levels were significantly upregulated in murine pulmonary fibrosis lungs and positively correlated with α-SMA and fibronectin expression, emphasizing its essential role in IPF pathogenesis and progression. GSEA further underscored KDM6B’s central function in modulating pathways associated with IPF, offering novel insights into the pathogenesis and potential treatment strategies for this disease. Our findings present compelling evidence that KDM6B and its associated molecules may serve as crucial modulators in IPF pathogenesis, and provide valuable insights into the underlying mechanisms of this disease.

## Data availability statement

The datasets presented in this study can be found in online repositories. The names of the repository/repositories and accession number(s) can be found in the article/**Supplementary Material**.

## Ethics statement

The animal study was reviewed and approved by Institutional Animal Care and Use Committee.

## Author contributions

GQ, JZ, HF and AC conceptualized the study. AC, ZS, and DS collected the transcriptome and clinical data, conducted experiments, and participated in data analysis. MH provided assistance in performing the experiments. GQ and AC drafted the manuscript. GQ, JZ, and HF revised the final manuscript. All authors contributed to the article and approved the submitted version.

## Supplementary material

Supplementary Figure 1 Validation of immune infiltration in a bleomycin((BLM)-induced pulmonary fibrosis mouse model. **(A)** Quantification of total macrophages, CD3^+^ T cells, and CD4^+^ T cells in lung tissue samples from PBS- or BLM-treated mice. The aggregated data from two independent experiments are presented. *n* = 6-8. Differences between the two groups were evaluated. **(B)** Relative proportions of macrophages, CD3^+^ T cells, and CD4^+^ T cells in lung tissue samples from PBS- or BLM-treated mice. The combined data from two independent experiments are displayed. *n* = 6-8. **(C)** Correlation analysis between KDM6B and CD4 expression in BLM-treated mice. **P* < 0.05, ***P* < 0.01, ****P* < 0.001. Data are represented as mean ± SEM.
