# Supplementary figures and images for: Integrative bioinformatics and validation studies reveal KDM6B and its associated molecules as crucial modulators in Idiopathic Pulmonary Fibrosis
Source: Front Immunol. 2023 May 19;14:1183871. doi: 10.3389/fimmu.2023.1183871 (PMC10235501; doi:10.3389/fimmu.2023.1183871)

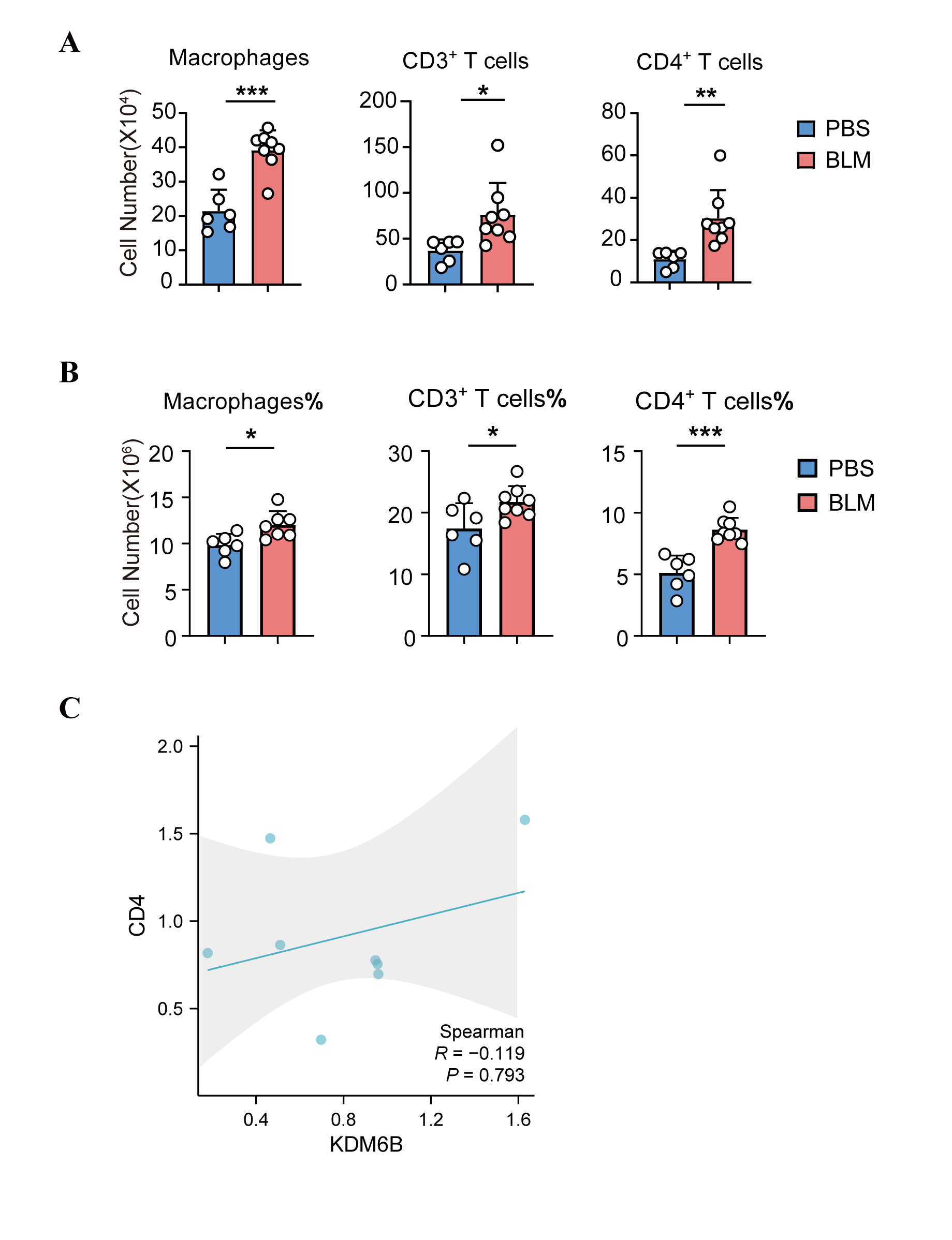

Supplement: Supplementary Figure 1 — Validation of immune infiltration in a bleomycin((BLM)-induced pulmonary fibrosis mouse model. (A) Quantification of total macrophages, CD3+ T cells, and CD4+ T cells in lung tissue samples from PBS- or BLM-treated mice. The aggregated data from two independent experiments are presented. n = 6-8. Differences between the two groups were evaluated. (B) Relative proportions of macrophages, CD3+ T cells, and CD4+ T cells in lung tissue samples from PBS- or BLM-treated mice. The combined data from two independent experiments are displayed. n = 6-8. (C) Correlation analysis between KDM6B and CD4 expression in BLM-treated mice. *P < 0.05, **P < 0.01, ***P < 0.001. Data are represented as mean ± SEM. [file Image_1.tif]
